# Dalbavancin for Acute Bacterial Skin and Skin Structure Infections in Pediatrics: Insights from Continuation Therapy Experience

**DOI:** 10.3390/antibiotics13040327

**Published:** 2024-04-04

**Authors:** Sara Maria Scarano, Eugenia Bruzzese, Marco Poeta, Margherita Del Bene, Alfredo Guarino, Andrea Lo Vecchio

**Affiliations:** 1 Pediatric Infectious Disease Unit, Department of Maternal and Child Health, University Hospital “Federico II”, 80131 Naples, Italy; saramaria.scarano@unina.it (S.M.S.); eugbruzz@unina.it (E.B.); marco.poeta@unina.it (M.P.); margherita.delbene@gmail.com (M.D.B.); alfguari@unina.it (A.G.); 2 Department of Translational Medical Science, University of Naples “Federico II”, 80138 Naples, Italy

**Keywords:** dalbavancin, bacterial infection, skin infection, soft tissue infection, ABSSSI, children

## Abstract

Acute Bacterial Skin and Skin Structure Infections (ABSSSI) are marked by substantial morbidity, frequent need for hospitalization, and long courses of intravenous antibiotic therapy. Herein, we report four cases of pediatric patients admitted for ABSSSI and managed with a combination antibiotic regimen incorporating dalbavancin: a second-generation lipoglycopeptide active against Gram-positive bacteria, including methicillin-resistant *Staphylococcus aureus*. In our experience, particularly in a setting with a high methicillin-resistance rate, dalbavancin demonstrated safety and efficacy, simplifying ABSSSI management in childhood. Its prolonged half-life enables a single-dose administration regimen, offering potential solutions to numerous challenges encountered in pediatric care, such as extended hospital stays, difficulties in securing and maintaining vascular access, lack of pediatric-specific drug indications, and limited availability of suitable oral formulations.

## 1. Introduction

According to the definition provided by the Food and Drug Administration, acute bacterial skin and skin structure infections (ABSSSI) encompass a range of complex skin and soft tissue infections, such as erysipelas, cellulitis, wound infections, and skin abscesses, characterized by extensive lesions covering a minimum area of 75 cm^2^ [1].

While the definition of complicated abscess in children typically relies on such parameters as age-specific maximum diameter, the number of lesions, and potential recurrence [2], a specific size cutoff for ABSSSI has yet to be established within the pediatric population.

These infections present a prevalent and clinically consequential challenge in pediatric patients, marked by notable morbidity, frequent need for hospitalization, and prolonged courses of intravenous antibiotic therapy. Over recent years, there has been an increase in hospitalization rates for ABSSSI, coinciding with the emergence and spread of methicillin-resistant *Staphylococcus aureus* (MRSA) strains [3,4] and a surge in the epidemiology of *Streptococcus pyogenes* infections in childhood [5].

Although there are multiple therapeutic approaches available for treating pediatric ABSSSI, managing these conditions is often challenging due to several barriers. These include the necessity for prolonged hospital stays in severe cases, the absence of pediatric indications for certain drugs, a restricted availability of oral formulations suitable for children, and the increasing prevalence of antibiotic resistance even among community-acquired bacterial strains. These barriers have recently prompted research and medical attention towards new therapeutic options.

Here, we present four cases of pediatric patients hospitalized for ABSSSI and managed with therapeutic regimens based on dalbavancin, offering valuable insights into the utilization of long-acting antibiotics in childhood (Table 1).

## 2. Cases Presentation

### 2.1. Patient #1

A 4-year-old child with autism spectrum disorder presented with fever and an erythematous, edematous, and painful lesion on the right calf with a maximum diameter of 4 cm and dimensions of approximately 20 cm^2^, clinically attributable to cellulitis. Due to the difficulties in oral administration, poor clinical response to amoxicillin-clavulanate, and the finding of neutrophilic leukocytosis with elevated inflammatory markers (C-reactive protein 71 mg/L and procalcitonin 2.0 ng/L), the child was hospitalized for further diagnostic evaluation (negative blood cultures) and initiation of intravenous treatment with clindamycin. After 72 h of antibiotic therapy, there was an initial clinical improvement with defervescence, reduction in inflammatory markers, decreased local warmth, and a 30% reduction in the surface extension of skin lesion.

However, during the hospitalization, significant management difficulties arose due to the child’s underlying conditions. His hyperactive and occasionally self-injurious behavior led to frequent removal of the venous access (five times in the first 48 h), and he barely accepted hospital stay and related health-care procedures. Considering the challenges with either oral and intravenous antibiotic regimens, which constituted the sole reason for hospitalization, a single dose of dalbavancin (administered at a dosage of 22.5 mg/kg) was administered, and the child was discharged with scheduled weekly follow-up appointments. Two weeks later, clinical and laboratory checks showed the resolution of cellulitis, full normalization of hematological parameters, and absence of adverse events.

### 2.2. Patient #2

**Figure 1:**
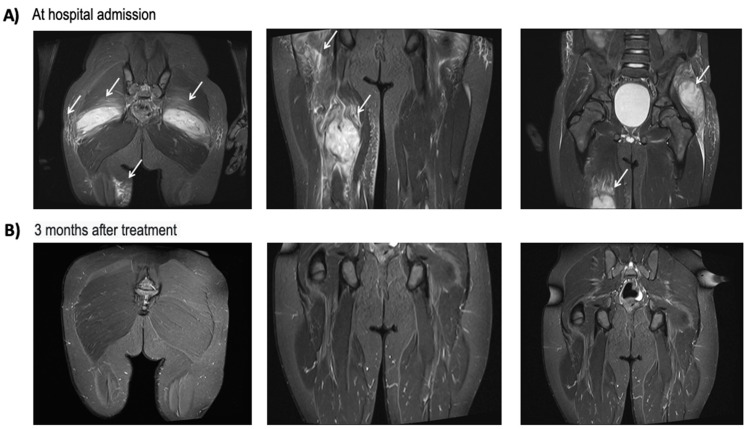
Imaging of subcutaneous and muscular abscesses caused by PVL-positive MRSA infection. Magnetic resonance imaging (MRI) scans of a 9-year-old child (Patient #2) admitted for multiple bilateral subcutaneous and muscular abscess collections (white arrows) localized to the thigh and gluteal region, attributed to PVL-positive *S. aureus* (Panel **A**). The patients underwent targeted antibiotic treatment for 3 weeks followed by a single infusion of dalbavancin. Three months post-treatment, a new MRI (Panel **B**) revealed complete resolution of subcutaneous and muscular lesions.

A 9-year-old presented with poor clinical conditions, fever, and limping. Recent history revealed frequent and intense physical activity with mild but recurrent traumatic events (i.e., quad bike racing, springboard diving). Hematological tests revealed neutrophilic leukocytosis and a sustained increase in inflammatory markers (C-reactive protein 303 mg/L). Magnetic resonance imaging (MRI) showed the presence of multiple and bilateral subcutaneous abscess collections, which extended to the posterior and anteromedial compartment of the thigh bilaterally and to the gluteal region (Figure 1).

During the hospital stay, a congenital deficiency of factor VII was diagnosed. This finding may explain the occurrence of multiple bilateral hematomas affecting the muscles of the lower limbs, potentially predisposing the child to local superinfection and subsequent development of abscesses. Blood cultures were sterile, while the culture of material drained from the abscess revealed the presence of MRSA producing Panton–Valentine leucocidin (PVL), resistant to oxacillin (MIC > 2 mcg/mL) and clindamycin (MIC = 0.5 mcg/mL), susceptible to levofloxacin at increased exposure (MIC = 1 mcg/mL) and susceptible to rifampicin (MIC ≤ 0.03 mcg/mL) and linezolid (MIC = 2 mcg/mL). Due to the lack of clinical response, the first-line antibiotic treatment was replaced with linezolid and rifampicin, along with surgical drainage of the larger abscess lesions. This treatment resulted in the resolution of fever, a significant reduction in inflammatory markers, and notable improvements in both clinical symptoms and radiological findings. After three weeks of intravenous antibiotic therapy and normalization of inflammatory markers, the patient was clinically dischargeable. Following discussion with the family regarding therapeutic options and considering the challenges associated with prescribing an off-label home treatment, such as linezolid, a single infusion of dalbavancin (administered at a dosage of 18 mg/kg) was administered prior to the patient’s discharge. Additionally, due to the isolation of PVL, oral rifampicin was prescribed for home administration. Two weeks post-discharge, the child presented good clinical conditions, reporting neither difficulties walking nor clinical side effects. At three months, the MRI findings appeared normalized.

### 2.3. Patient #3

A 2-year-old girl was admitted to the hospital with fever and a painful skin lesion of about 60 cm^2^ in the left chest region, characterized by the presence of erythema, edema, and a central area undergoing colliquation. The diagnostic work-up demonstrated a neutrophilic leukocytosis with elevated inflammatory markers (C-reactive protein 101 mg/L and procalcitonin 1.0 ng/L) and negative blood cultures. A PVL-positive MRSA (MIC oxacillin > 2 mcg/mL) susceptible to clindamycin (MIC = 0.25 mcg/mL) and rifampicin (MIC ≤ 0.03 mcg/mL) and at increased exposure to levofloxacin (MIC = 1 mcg/mL) was isolated by the drainage of the central necrotic lesion. A good clinical and laboratory response under an empiric intravenous therapy with clindamycin allowed an early shift (after 4 days) to oral treatment. However, she showed poor compliance with oral therapy; hence, we decided to administer a single dose of dalbavancin (22.5 mg/kg) and to discharge at home. Three weeks later, she was clinically recovered, and no adverse events were reported.

### 2.4. Patient #4

A 2-year-old girl presented with fever and a painful skin lesion exhibiting erythema and edema, which extended throughout her entire lower limb and worsened following the initiation of oral therapy with amoxicillin-clavulanic acid, a regimen that was also administered with poor adherence. In addition, she exhibited severe atopic dermatitis, likely predisposing her to the infection. The diagnostic work-up did not identify any etiological agent, but the patient showed a good response to intravenous clindamycin treatment, showing negative inflammatory markers, defervescence, and improvement of the skin lesion after 3 days. Given the previously demonstrated poor adherence to oral therapy, we decided to administer a single dose of dalbavancin (22.5 mg/kg) and to discharge her at home. Two weeks later, she had clinically recovered with no reported adverse events.

## 3. Discussion

Since December 2022, the European Medicines Agency has extended the indication to dalbavancin use to children as young as 3 months old with ABSSSI [6]. Dalbavancin is a second-generation lipoglycopeptide with bactericidal activity, which inhibits bacterial cell wall synthesis by binding to the D-alanyl-D-alanine terminus of the peptidoglycan. The in vitro spectrum includes clinically relevant Gram-positive bacteria, including *Streptococcus pyogenes*, the *Streptococcus viridans* group, vancomycin-sensitive *Enterococcus faecalis*, and both methicillin-sensitive and resistant *Staphylococcus aureus*, against which it has demonstrated 32-fold greater potency than vancomycin [7]. Resistance to dalbavancin among Gram-positive bacteria is currently limited to those expressing the VanA phenotype [6,7]. Recent evidence also demonstrates the ability of dalbavancin to penetrate and act on biofilm, a mechanism of pathogenicity and resistance exerted by Gram-positive species, such as Staphylococci and Enterococci [8,9].

From a healthcare perspective, the most intriguing aspect is the pharmacokinetic profile. The high drug–protein binding and extensive volume of distribution give dalbavancin a half-life exceeding 15 days (range 333–404 h), allowing a single intravenous administration regimen [6,10]. Additionally, dalbavancin has limited interaction with other drugs, given the absence of cytochrome P450 induction or inhibition [6], which occasionally limit the efficacy and prescription of antibiotics included in the pipeline of drugs used for the management of skin and soft tissues infections (e.g., rifampicin).

The evidence supporting the approval of dalbavancin for pediatric ABSSSI treatment comes from a single multicenter randomized study in which 191 subjects under 18 years with known or suspected Gram-positive bacterial ABSSSI were allocated to three treatment groups: a single dose of dalbavancin (dose adjusted for weight and age, up to 1500 mg), two doses of dalbavancin (first dose adjusted for weight and age, up to 1000 mg, and second dose adjusted for weight, up to 500 mg one week later), or a comparator group (with the option to switch to oral cefadroxil or clindamycin). The study demonstrated excellent efficacy, with a reduction in skin lesions after 48–72 h post-infusion in almost all children treated with a single dose (97.4%) or a two-dose regimen (98.6%). The drug also showed an excellent safety profile, with no serious adverse reactions attributable to the drug and no ototoxicity, tested with audiometric evaluation in a subgroup of patients at the beginning of treatment and after 28 days [11].

The in vitro spectrum, along with the safety profile demonstrated by preliminary data, and notably the distinct pharmacokinetic characteristics, render this drug an optimal choice for treating pediatric ABSSSI.

Microbiological findings from cohorts of children with ABSSSI indicate *Staphylococcus aureus* as the main etiology, and MRSA strains, frequently community-acquired (CA-MRSA), have increased in recent years with rates close to 40% of isolates in pediatric patients [12]. This frequently forces practitioners in settings with elevated rates of antimicrobial resistance to transition from first-line antibiotic treatments, such as oxacillin, to alternative molecules effective against MRSA. In the Campania region of Southern Italy, where the children described in this study reside, the prevalence of MRSA reaches 35% (with invasive samples at 39%, respiratory samples at 30%, and other materials at 34%), while strains resistant to clindamycin approach 40% across all isolates [13].

Considering that CA-MRSA strains are often toxin producers, and that skin localization is the most frequent manifestation of infection in pediatric patients [14], the role of dalbavancin in the treatment of ABSSSI caused by PVL-producing *S.aureus* needs to be better studied. For such infections, the International Society of Chemotherapy suggests a combined antibiotic regimen incorporating agents with anti-toxin activity, such as protein synthesis inhibitors, particularly for invasive bone and lung infections. However, it seldom deems combination therapy essential for skin and soft tissue infections, except in cases of highly necrotizing forms [15]. In a recent in vitro study on over 300 *S. aureus* isolates, 40% of which were PVL producers, all strains were found to be sensitive to dalbavancin [16]. In our experience, two cases of PVL-producing *S. aureus* treated with a dalbavancin and rifampicin regimen showed a positive clinical outcome with no recurrences after over three months of follow-up.

For all described patients, the use of a long-acting drug helped to reduce the length of hospital stay and the days of treatment. The pharmacokinetic profile of dalbavancin limits the therapeutic regimen to one or two administrations rather than weeks of (often) intravenous therapy requiring hospital stay. Furthermore, long-acting treatment options not only result in reduced hospital stays, thus optimizing healthcare expenses, but also decrease the risk of nosocomial infections. Additionally, mitigating the discomfort associated with prolonged hospitalization, significantly contribute to the physical and psychological well-being of patients, especially in peculiar conditions (e.g., the child with autism) that hamper prolonged hospital stays. All of this has a direct impact on families in terms of quality of life and a reduction in workday loss. Preliminary findings indicate that 80% of families reported minimal interference with their child’s daily activities during dalbavancin therapy, contrasting with 47% of patients on alternative antibiotic regimens [11].

Moreover, simplification of intravenous antibiotic therapy reduces the need for obtaining and maintaining stable venous access. The latter is a challenge frequently overlooked by healthcare professionals handling adult patients but presenting notable hurdles in managing children and their families. The placement of suitable venous access, occasionally cumbersome in infants and preschool-aged children, induces discomfort to the patient and hastens the depletion of superficial venous reserves.

Additionally, prolonged antibiotic therapy required for treating certain forms of ABSSSI necessitates central venous access placement with the need for patient sedation, involvement of adequately trained personnel, and management of potential medium-term complications (e.g., dislocation, infections).

Lastly, the compliance of children with oral antibiotic therapies is often suboptimal due to the need of multiple daily administrations and the long duration of treatment. Furthermore, the lack of pediatric formulations for drugs frequently used in the management of ABSSSI is associated with poor palatability, which increases the risk of suboptimal dosages and, hence, worsens overall treatment adherence. In certain instances, such challenges coexist, as exemplified by the child with autism spectrum disorder mentioned previously, where the administration of both oral and extended intravenous medications posed significant obstacles.

Although the use of dalbavancin is approved only for ABSSSI treatment, the above-discussed aspects are likely applicable to many other infections caused by Gram-positive bacteria that often require an even longer antibiotic therapy, in which context a drug with long-acting efficacy could address an even more significant clinical need. Some off-label use evidence of dalbavancin in infections caused by *S. aureus*, such as arthritis, osteomyelitis, endocarditis, or prosthetic infections, reports favorable outcomes [17].

Caselli et al. recently reported a good safety and effectiveness in 31 Italian children receiving dalbavancin for the treatment of soft tissue and bone infections [18]. However, about a year after the approval of dalbavancin for pediatric use, available real-life usage evidence in children is quite limited. The primary obstacles to pediatric utilization likely stem from challenges in diagnosing the etiology, the existing availability of antibiotic molecules with established efficacy and safety records, and the simultaneous concern that potential adverse events of a new long-half-life drug could theoretically manifest with delayed onset (even at home) or prolonged duration. In our experience, we have employed dalbavancin to conclude ongoing antibiotic treatments that had already shown clinical and biochemical efficacy.

Future clinical research should consolidate the safety and efficacy data, produce real-life “effectiveness” evidence, test the differences in various therapeutic regimens, and expand usage indications to other pathologies, exploiting the drug’s pharmacokinetic peculiarities and possible biofilm activity. This would allow a broader application, even as a first-line therapeutic option, of a drug that has innovative potential for infections requiring long-term therapy, placing the needs of the child and their family at the center of the care process.

## Figures and Tables

**Table 1:** Cases summary.

	Patient #1	Patient #2	Patient #3	Patient #4
Clinical Characteristics				
Age (years)	4	9	2	2
Underlying conditions	Autism spectrum disorder	Congenital factor VII deficiency	None	Atopic dermatitis
WBC at admission (10^3^/mm^3^)	12.95	24.58	12.72	31.66
CRP at admission (mg/L)	71.0	221.7	72.3	77.2
Indication for antibiotic treatment	Cellulitis	Muscle abscesses	Cellulitis	Cellulitis
Disease localization	Right calf	Thigh and gluteal region	Chest	Right lower limb
Microbiological evaluation				
Blood culture or NAAT *	Negative	Negative	Negative	Negative
Nasal swab	Negative	NP	Negative	NP
Lesion sample (aspiration, evacuation)	NP	Positive	Positive	NP
Bacterial isolation	NP	PVL-MRSA	PVL-MRSA	NP
Primary antibiotic treatment	Clindamycin	Linezolid+ rifampicin	Clindamycin	Clindamycin
Dalbavancin administration				
Dosage (mg/kg)	22.5	18	22.5	22.5
Number of doses	1	1	1	1
Time from hospitalization (weeks)	1	3	1	1
Reason for dalbavancin administration	- Poor acceptance of hospital stay - Poor acceptance of venous access - Poor adherence to oral drugs	- Oral drugs available only as off-label therapy	- Poor adherence to oral drugs - Pediatric formulation not available	- Poor adherence to oral drugs - Pediatric formulation not available
WBC at dalbavancin infusion	9.71	6.57	8.99	10.89
CRP at dalbavancin infusion	5.95	1.26	0.6	4.86
Associated antibiotic treatment	None	Rifampin	Rifampin	None
Adverse events at administration	None	None	None	None
Adverse events after 2 months of follow-up	None	None	None	None
Clinical outcomes	Healed	Healed	Healed	Healed

Legend. NP: not performed, NAAT: Nucleic acid amplification test, MRSA: methicillin-resistant *Staphilococcus aureus.* * Duplex PCR for the research of *S. pneumoniae* and *Hemophilus influenzae* and a Multiplex PCR test that includes probes for the research of *Enterococcus faecium*, *S. aureus*, *Klebsiella pneumoniae*, *Acinetobacter baumanii*, *Pseudomonas aeruginosa*, and *E. coli*.

## Data Availability

The data presented in this study are available on request from the corresponding author.
